# Health Related Quality of Life of Cancer Patients in Ethiopia

**DOI:** 10.1155/2018/1467595

**Published:** 2018-04-15

**Authors:** Tadesse Melaku Abegaz, Asnakew Achaw Ayele, Begashaw Melaku Gebresillassie

**Affiliations:** College of Medicine and Health Sciences, School of Pharmacy, Department of Clinical Pharmacy, University of Gondar, Gondar, Ethiopia

## Abstract

**Background:**

Neoplasm, AKA cancer (Ca), is associated with major morbidity and mortality.

**Aim:**

Measurement of health related quality of life (HRQoL) of Ca patients is uncommon in Ethiopia. The present study determined the HRQoL and its determinants among people living with Ca in north Ethiopia.

**Methods:**

A prospective hospital based study was conducted from 1 January 2017 to 30 August 2017 on Ca patients attending cancer treatment center of University of Gondar Teaching Hospital. The European Organization for Research and Treatment of Cancer Questionnaire version 3 was utilized to collect the data. The rate of QoL was presented using means with standard deviation (±SD). Binary logistic regression was employed to determine factors associated with HRQoL.

**Result:**

The present study is based on the findings from 150 subjects. The rate of QoL was 52.7 (20.1) (mean ± SD). The highest functional status was emotional functioning 61 (25.5). Patients with no disease metastasis, 92.1 (5.1), had high QoL as compared to metastasis, 22.1 (18.9) (*p* = 0.03). Patients with affected physical functioning have a 20% reduction in QoL and Adjusted Odds Ratio (AOR) of 0.794 [0.299–891]. Patients with low satisfaction level with the provided care, 0.82 [0.76–0.93], and those with unmet needs, 0.85 [0.80–0.95], experienced reduced level of HRQoL.

**Conclusion:**

Health related quality of life of cancer patients was found to be low in Ethiopia. Patients with limited rate of disease metastasis had improved HRQoL. Further, the unmet needs of Ca patients and the level of satisfaction with the overall care were found to influence the extent of HRQoL. Therefore, early detection of neoplasm to arrest metastasis is warranted in order to achieve better QoL. In addition, addressing the unmet needs of these patients and ensuring higher satisfaction rate are recommended to maintain adequate HRQoL.

## 1. Introduction

Neoplasm, AKA cancer (Ca), is associated with major morbidity and mortality in the world. A twenty-five-year systematic analysis of cancer registry from 195 countries demonstrated that there were 17.5 million cancer cases and 8.7 million deaths in the year 2015 worldwide [1]. Over a ten-year period (2005–2015), cancer cases increased by one-third (33%). The longevity of the general population (16%) and population density (13%) contributed to this magnitude. Breast cancer is the most commonly isolated cancer and the leading cause of mortality among women. Globally, it is attributed to one-fourth of the total cancer diagnosis and 14% of cancer deaths. Lung cancer is the leading malignancy site in males and makes 17% of the total new cancer incidence and 23% of the gross cancer deaths [2]. In Africa, cancer mortality was estimated to be 542,000 with a diagnosis of 715,000 new cancer cases as of 2008 [3].

Cancer is emerging as a formidable challenge in low income countries that have limited logistic to protect the health of citizens. In developing countries, the burden of Ca overlaps with the magnitude of infectious diseases including HIV/AIDS, tuberculosis, hepatitis virus, and human papilloma virus which can contribute to the pathogenesis of Ca. The lack of early detection and timely treatment would aggravate the situation in these nations [4]. In Ethiopia, cancer belongs to the second most common noncommunicable disease (NCD) only next to cardiovascular disorders [5]. In Gondar University Hospital, the number of new cases seen has increased following the establishment of new cancer treatment center. Tefera et al. 2016 reported that the top three cancer types were lymphoma (17.2%), cervical cancer (15.2%), and breast cancer (14.1%), respectively [6].

While treating cancer patients, we usually set different end points to measure the effectiveness of our intervention. Some of the parameters are regarded as primary end points and coprimary and surrogate (intermediate) endpoints. These measurements include Overall Survival (OS), Progression-Free Survival (PFS), Overall Radiographic Response (ORR), and health related quality of life (HRQoL). OS is an objective primary endpoint which measures all causes of death. But, it does not determine the exact impact of treatment [7]. PFS is an intermediate end point which predicts OS rate within short period of time and with reduced cost. However, PFS does not imply the clinical advantage of the treatment for the patient since the PFS is achieved in the expense of treatment toxicity and decline in HRqol. Like PFS, ORR directly measures the extent of the tumor through radiography. Nonetheless, it lacks reproducibility due to observer bias. Moreover, the above measures do not incorporate the patient perspective. On the other hand, HRqol is self-perceived approach to evaluate patients' view of their own health status [7]. The definition of HRqol remains different among different literatures. HRQoL can be defined as “how well individuals function on some predefined activities in their life and wellbeing in physical, mental, and social domains of health.” Wellbeing refers to an individual's subjective feelings [7, 8]. It is assessed by a standard structured questionnaire called Quality of Life Questionnaire (QLQ) prepared by the European organization for research and treatment of cancer. It has been used for clinical trials. But, recently it is introduced in nontrial studies [9, 10].

The significant number of people living with cancer (PLWCa) rarely achieves reemission with chemotherapy, surgery, or radiotherapy. In these patients, our goal is to improve their quality of life and to promote their functioning. HERqol is an important tool used to evaluate the functioning of our patients. Assessment of HERqol is also helpful to pass shared decision between the patient and the clinician regarding the treatment. But, it has not been implemented in our setup so far. Therefore, the present study aimed to investigate cancer patients' health related quality of life at Gondar University Hospital Cancer Center.

## 2. Methods

### 2.1. Study Setting and Area

University of Gondar Referral Hospital (UoGRH) is a teaching hospital located in Gondar Town, northwest Ethiopia. Gondar is 748 kms away from the capital, Addis Ababa. The Gondar Cancer Center was established in January 2015 with few dedicated individuals. It is regarded as the second treatment center in the country. More than 600 patients visit the center for chemotherapy and screening. It is run by few physicians and nurses who have gained adequate training on the discipline. The inpatient ward contains ten beds in which chemotherapy is administered in each cycle. The cytotoxic admixture and administration are carried out by nurses. However, there is no radiotherapy service in the hospital so far.

### 2.2. Population

All cancer patients who were admitted to oncology ward of UoGRH during the study period were our source populations, whereas patients above the age of 18 years were the study population.

### 2.3. Study Design and Period

A prospective hospital based cross-sectional study was conducted from 1 January 2017 to 30 August 2017.

### 2.4. Inclusion and Exclusion Criteria

Patients who were receiving therapy and are above 18 years old were included. Those who did not consent for the study and are unable to respond for the questions were excluded.

### 2.5. Sample Size Determination and Sampling Technique

All cancer patients were consecutively included in the study based on inclusion and exclusion criteria during the study period.

### 2.6. Study Variables

Our dependent variable was the rate of HRQoL. Independent variables include sociodemographic characteristics of the patient including age and gender, functional status, and symptom scales.

### 2.7. Data Collection Methods

Data was collected by two trained clinical nurses. A structured questionnaire which contained of 30 items was adopted from the Quality of Life Questionnaire (QLQ-30) version 3 which is the standard version currently. It was released in 1993 [9]. The questionnaire contains five multi-item functional status scales (physical, role, social, emotional, and cognitive) and 9 symptoms scales (pain, fatigue, financial impact, appetite loss, and nausea/) and two global health status items.

The scoring of the findings was based on EORTC QLQ-30 scoring manual [10, 11]. The scales are rated in terms of percentage. A high score in functional scale and global health status denotes high health status, respectively. But, for a symptom scale high score represents sever symptomatology.

### 2.8. Data Quality Control Technique

Data collectors were trained intensively on contents of the questionnaire, data collection methods, and ethical concerns. The questions were translated into Amharic so as to maintain unbiased response. The filled questionnaire was checked daily for completeness by the principal investigator. The reliability (psychometric property) of the tool was evaluated and demonstrated a Cronbach alpha value of 0.871. The content of the questionnaire was reviewed by senior experts.

### 2.9. Data Analysis

All the statistical data were carried out using Statistical Package for Social Sciences (SPSS), version 20 (SPSS Inc., Cary, NC, USA). Descriptive statistics was presented using means with standard deviation (±SD) and percentages (%). *p* values were kept <0.05 with 95% confidence interval. Bivariate analysis was applied to investigate the correlation of independent variables. Binary logistic regression was employed to determine associated factors. One-way analysis of variance has been employed to assess the mean difference in quality of life.

## 3. Results

### 3.1. Sociodemographic and Clinical Characteristics of Patients

The present study was based on the findings from 150 subjects. All of the patients who attended the cancer treatment center responded to the questionnaire. The mean age of the respondents was 46.8 (14.5). More than half of the patients were females 83 (52.9%). Above forty percent of them did not have formal education 69 (43.9%). The average monthly income was 1336.1 ± 240.3 Ethiopian birr ($49.48 ± 8.9, $ = 27.175 ETB). Nearly forty percent of cases were metastasis 65 (41.4). The mean duration of the disease was 13.4 ± 12.1 months. The most common Ca include breast Ca 37 (24.7) followed by blood related Ca 36 (24). The frequently prescribed medications include leucovorin (79), 5-fluorouracil (68), and cisplatin (47), respectively (Table 1).

### 3.2. Global Health Status, Functional Scales, and Symptom Scales

The rate of quality of life based on global health status (GHS) was 52.7 (20.1). The highest FS was emotional functioning (EF) 61 (25.5) followed by cognitive functioning 59.31 (43.6%). The physical functioning state of the patients was 53.27 (22.9) whereas social functioning (SF) and role functioning (RF) accounted to 46.31 (25.5) and 43.32 (26.7), respectively. Nausea and vomiting were the most annoying symptom, 43.3 (23.1) followed by 42.1 (33.3), and fatigue, 41.47 (24.5) (Table 2).

### 3.3. The Mean Difference in QoL Scales versus Sociodemographic Characteristics

RF was found to be different based on marital status (*p* = 0.02). According to post hoc analysis, the difference was found to be between single marital status, 43 (31.6), and divorced, 1.2 (2.3) (*p* = 0.01), and married, 36.7 (29.8), and divorced (*p* = 0.03). The mean difference of GHS was significant for disease metastasis. Accordingly, patients with no disease metastasis, 92.1 (5.1), had high GHS as compared to metastasis, 22.1 (18.9) (*p* = 0.03). The mean difference of EF was also significant in terms of disease metastasis, 34.1 (25.5) versus 53.2 (25.3) (*p* = 0.04). GHS was also different for patients who underwent surgical procedure, 82.8 (4.9) and 24.5 (19.5) (*p* = 0.01) (Table 3).

### 3.4. Multinomial Regression Indicating Factors Affecting Quality of Life

Binary logistic regression indicated that patients with affected physical functioning have a 20% reduction in quality of life of AOR of 0.794 [0.299–891]. Patients with no history of vomiting were 2.5 more likely to have good QoL as compared to patients with vomiting history of AOR of 2.655 [1.839–8.397]. Patients whose social functioning is not affected more than three times are more likely to have good QoL of 3.637 [1.838–8.300]. Patients with unaffected EF had 4.5 times good HRQoL as compared to affected HRQoL of 4.426 [2.890–6.613]. Patients with low satisfaction level with the provided care of 0.82 [0.76–0.93] and those with unmet needs of 0.85 [0.80–0.95] experienced reduced level of HRQoL (Table 4).

## 4. Discussion

Ca and its treatment strategies substantially affect HRQoL of patients. HRQoL is viewed as one of treatment end points in these individuals. Estimation of HRQoL of patients living with Ca helps to evaluate the effectiveness of our interventions. In developing countries including Ethiopia, HRQoL measurement is not performed routinely. This study aimed to determine the rate of HRQoL among Ca patients attending a teaching referral hospital in north Ethiopia. Based on GHS data, the rate of quality of life was found to be 52.7 (SD: 20.1) which was comparable from a result obtained in Addis Ababa, 52.5 (SD: 26.0) [11], but lower from the reference value [12]. In addition, QoL was quite small when compared with other studies from India, Melbourne, Nepal, and Brazil [13–15]. Low level of QoL in our study might be due to quality of care provided in the setup. The cancer center has been established only recently, as of 2015, and advanced treatments including radiotherapy, adequate surgical procedure, and palliative care are yet to be started. In addition, patients are usually admitted once they are terminally ill. One study reiterated that level of care affects QoL [12]. Surgery has been linked with the improvement in QoL of patients in our study which is demonstrated by mean difference in QoL among individuals who underwent surgery.

The present study discovered that emotional and cognitive functions were among the highest functional status scores. They remain relatively unaffected. Emotions contain depression, worries, tension, and irritability whereas cognition evaluated the patients' level of concentration on things and their ability to remember. Patients report “not at all or a little” disturbance of emotion. Binary regression indicated that individuals with intact emotion were considered to have good QoL. In addition, a retrospective study in USA reported high cognitive functioning but low role functioning among hepatic Ca patients [16]. Rather, role functioning aspects such as doing daily activities and leisure were highly affected.

The most common compliance on symptom scale was nausea and vomiting followed by fatigue. Nauseas and vomiting are common in Ca patients due to the disease and therapy. The underutilization of antiemetics due to cost and nonadherence to guidelines might contribute to the prevalence of nauseas and vomiting [17]. Furthermore, fatigue was found to be the second most disabling symptom among Ca patients. It is resulted from the therapy including radiotherapy and chemotherapy as well as the disease state. Consequently, the QoL of patients is reduced as fatigue becomes sever [18]. Advanced disease states and the occurrence of psychosocial symptoms could aggravate the prevalence and severity of fatigue. Researches revealed that malignancy by itself could induce malaise and weakness [19].

The rate of role functioning was found to be different with respect to marital status. Single and married individuals had good role functioning as compared to divorced individuals (*p* < 0.05). Married persons tend to present early before metastasis and receive advanced care unlike other individuals. Other study also estimated that cancer survival rate was also affected by marital status. A comparative study indicated that widowed patients were found to be at greater risk of death relative to other groups [20]. With regard to GHS, the mean difference of GHS was significant based on the level of disease metastasis. Advanced diseases were found to reduce the GHS and emotional functioning of Ca patients. Accordingly, patients with no disease metastasis had high GHS as compared to metastasis (*p* = 0.03). Another finding on this study indicated that surgery showed a positive impact on the global health status of patients since it could bring a radical cure of the diseases if it is followed by adequate adjuvant therapy. But, it is difficult to generalize this finding for all forms of Ca as some cases favor improved quality of life when surgery preserves organs such as breast cancer and lung cancer so as to spare aesthetic values [21–23].

In the current study, multiple factors have been correlated with QoL of cancer patients. It was found that patients with affected physical functioning have a reduced quality of life. The global health QoL and functional status of cancer patients usually go parallel. For instance, a study measured the oropharyngeal neoplasia and its function and the global health status demonstrated that patients with limited or compromised oropharyngeal function were having poor QoL [24]. Patients with no history of vomiting were 2.5 more likely to have good QoL as compared to patients with vomiting history. Vomiting was found to affect routine activities of patients including household activities, feeding style, time allocation for social activities, and daily function and recreation [25]. In addition, patients with preserved social functioning are nearly four times more likely to have good QoL. Furthermore, patients with unaffected EF had 4.5 times good HRQoL as compared to affected HRQoL. A comparative study reported that emotional disturbance among cancer patients could lead to low level of global QoL [26, 27].

In general, the present study provided baseline information on the quality of life of Ca patients in developing country. However, it is limited to single institution as well as few sample size. In light of this, large studies are recommended to increase the generalizability of findings.

## 5. Conclusion

Health related quality of life of cancer patients was found to be low in Ethiopia. Patients with limited rate of disease metastasis had improved HRQoL. Further, the unmet needs of Ca patients and the level of satisfaction with the overall care were found to influence the extent of HRQoL. Therefore, early detection of neoplasm to arrest metastasis is warranted in order to achieve better QoL. In addition, addressing the unmet needs of these patients and ensuring higher satisfaction rate are recommended to maintain adequate HRQoL.

## Figures and Tables

**Table 1:** Sociodemographic and clinical characteristics of cancer patients attending UoGRH.

Variables	*N* (%)
Age	46.8 (14.5)
Females	83 (52.9)
Occupation	
Nongovernmental	14 (8.9)
Private employee	36 (22.9)
Government employee	20 (2.7)
Agriculture	56 (37.33)
Retire	24 (16)
Education	
No education	69 (43.9)
Elementary	32 (20.4)
High school	25 (16.7)
College	12 (8)
University	12 (8)
Marital status	
Single	33 (21)
Married	83 (52.9)
Divorced	14 (8.9)
Widowed	20 (12.7)
Monthly income	1336.1 ± 240.3
Residence	
Rural	78 (49.7)
Surgery (yes)	72 (45.5)
Duration of the disease (months)	13.4 ± 12.1
Metastasis (yes)	65 (41.4)
Diagnosis	
Colorectal Ca	30 (20)
Cervical Ca	32 (21.33)
Lung Ca	15 (10)
Blood related Ca	36 (24)
Breast Ca	37 (24.7)
Medications	
5-Fluorouracil	68
Cisplatin	47
Leucovorin	79
Cyclophosphamide	41
Doxorubicin	39
Methotrexate	45
Irinotecan	27
Others	31

**Table 2:** The mean global health status, functional scales, and symptom scales of cancer patients at UoGRH.

Scales	mean ± SD
Global health status	52.7 (20.1)
Functional scales	
Physical functioning	53.27 (22.9)
Role functioning	43.32 (26.7)
Social functioning	46.31 (25.5)
emotional functioning	61 (25.5)
Cognitive functioning	59.31 (43.6)
Symptoms scale	
Fatigue	41.47 (24.5)
Nausea and vomiting	43.3 (23.1)
Pain	34.8 (24.4)
Dyspnea	34.8 (29.2)
Insomnia	42.1 (33.3)
Appetite	38.4 (31.2)
Constipation	40.6 (31.2)
Diarrhea	44.2 (34.1)
Financial difficulties	69.6 (31.2)

**Table 3:** The mean difference QLQ scales versus sociodemographic characteristics.

	QoL	PF	RF	EF	CF	SF
Marital status						
Single	26.9 (22.7)	52.6 (23.7)	43 (31.6)	47.1 (28.2)	56.5 (20)	44.9 (24.1)
Married	73.7 (4.6)	53.8 (24.4)	36.7 (29.8)	36.7 (25.1)	61.3 (54.8)	48.3 (25.9)
Divorced	26.3 (16.7)	53 (18.8)	1.2 (2.3)	38.9 (23.1)	63 (31.4)	36.9 (23.7)
Widowed	25 (20.5)	52.3 (18.1)	20 (29.1)	37.3 (22.9)	53.3 (22.1)	46.7 (27.4)
*p* value	0.871	0.989	**0.02 **	0.25	0.85	0.475
Sex						
Female	74.7 (4.6)	50.3 (23.4)	48.2 (29.9)	37.6 (24.4)	56 (24.1)	71.5 (29.1)
Male	25 (20.2)	56.9 (21.8)	37.3 (27.8)	41.3 (26.8)	63.4 (59.5)	67.2 (33.7)
*p* value	0.377	0.673	0.265	0.436	0.124	0.397
Education						
No education	80.8 (4.9)	51.7 (24.9)	50.7 (10.8)	36.1 (23.1)	57.2 (25.2)	44.9 (25.3)
Elementary	33.1 (19.3)	48.4 (21.5)	38.1 (31.1)	37.2 (26.1)	48.5 (24.8)	47.4 (19.3)
High school	25.9 (18.9)	59.4 (20.9)	37.3 (35.3)	48.7 (30.8)	74.9 (65.3)	41.6 (30.1)
College and above	22.9 (14.7)	57.6 (19.1)	35.4 (23.1)	23.8 (49.7)	63 (19.4)	55.7 (24.4)
*p* value	0.361	0.152	0.57	0.175	0.128	0.214
Residence						
Urban	24.1 (17.5)	54.9 (23.5)	50.4 (10.3)	40.3 (24.7)	60 (25.2)	47 (25.7)
Rural	84.1 (3.4)	51.4 (22.1)	35.6 (28.3)	38.1 (26.4)	58.5 (57.4)	45.6 (25.5)
*p* value	0.283	0.341	0.239	0.608	0.836	0.73
Duration of the disease						
0–12 months	65.5 (4.1)	52.4 (22.6)	45.7 (19.1)	40.6 (26.2)	57.5 (25.1)	44.1 (25.71)
13-24	29.6 (22.2)	54.2 (22.9)	39.4 (35.9)	31.7 (21.7)	65.1 (81.3)	48.9 (26.0)
≥25	16.7 (13.7)	57.5 (25.3)	35.5 (30)	46.4 (25.8)	58.8 (21.8)	55.5 (21.50)
*p* value	0.412	0.531	0.162	0.11	0.689	0.217
Metastasis						
Yes	22.1 (18.9)	49.9 (19.5)	34.1 (27.1)	34.1 (25.5)	54.6 (22.7)	45.1 (27.7)
No	92.1 (5.1)	55.8 (24.9)	50.34 (29.8)	53.2 (25.3)	62.9 (54.3)	47.2 (23.8)
*p* value	**0.03**	0.11	0.20	**0.04**	0.246	0.625
Surgery						
Yes	82.8 (4.9)	53.4 (20.7)	36.4 (30.8)	37.4 (25.6)	61.6 (57.2)	48.1 (25.1)
No	24.5 (19.5)	53.2 (24.8)	49.7 (10.2)	40.95 (25.4)	57.2 (25.6)	44.6 (25.9)
*p* value	**0.01 **	0.96	0.289	0.39	0.545	0.41

PF: physical functioning, RF: role functioning, EF: emotional functioning, CF: cognitive functioning, and SF: social functioning.

**Table 4:** Association between functional and symptom scales and quality of life.

Variables	QoL		COR 95% CI	AOR 95%
	Affected	Not affected		
Sex				
Male	21 (14)	22 (14.7)	1	1
Females	61 (40.7)	46 (30.7)	1.34 [0.648–2.713]	1.419 [0.596–3.378]
Age	47.3 (13.6)	45.7 (14.9)	1.11 [0.98–1.21]	1.002 [0.971–1.034]
Residence				
Rural	57 (38)	21 (14)	0.877 [0.429–1.79]	1.482 [0.630–3.484]
Urban	50 (33.33)	22 (14.7)	1	1
Duration of the Dz (month)	11.91	13.27	0.11 [0.97–1.1]	1.001 [0.959–1.045]
Surgery				
No	58 (38.7)	20 (13.33)	1.433 [0.7–2.95]	1.084 [0.447–2.633]
Yes	49 (32.7)	23 (15.33)	1	1
Metastasis				
Yes	49 (32.7)	24 (16)	0.728 [0.351–1.511]	0.590 [0.235–1.479]
No	16 (10.7)	26 (17.33)	1	1
Physical functioning				
No	61 (40.7)	27 (18)	1	1
Yes	46 (30.7)	16 (10.7)	1.357 [0.649–2.84]	0.794 [0.299–891]^*∗*^
Role functioning				
Yes	42 (28)	21 (14)	0.646 [0.315–1.33]	0.655 [0.220–1.949]
No	65 (43.3)	21 (14)	1	1
Emotional functioning				
Yes	46 (30.7)	14 (9.33)	1	1
No	61 (40.7)	29 (19.3)	1.682 [0.788–3.59]	4.426 [2.890–6.613]^*∗*^
Cognitive functioning				
Yes	36 (24)	9 (6)	1	1
No	71 (47.3)	34 (22.7)	2.56 [0.91–5.14]	2.286 [0.684–7.637]
Social functioning				
Yes	36 (24)	7 (4.7)	1	1
No	71 (47.3)	36 (24)	3.04 [1.173–7.89]	3.637 [1.838–8.300]^*∗*^
Fatigue				
Yes	57 (38)	14 (9.3)	2.45 [1.15–5.25]	1.999 [0.488–8.188]
No	50 (33.3)	28 (18.7)	1	1
Vomiting				
Yes	55 (36.7)	18 (12)	1	1
No	52 (34.7)	25 (16.7)	1.55 [0.756–3.21]	2.655 [1.839–8.397]^*∗∗*^
Pain				
Yes	54 (36)	28 (18.7)	1	1
No	53 (35.33)	15 (10)	0.529 [0.251–1.14]	0.639 [0.217–1.881]
Dyspnea				
Yes	36 (24)	20 (13.33)	0.614 [0.296–1.271]	0.867 [0.308–2.438]
No	71 (47.33)	23 (15.33)	1	1
Appetite				
Yes	38 (25.33)	11 (7.33)	1	1
No	69 (46)	32 ( 21.33)	1.762 [0.782–3.973]	1.784 [0.487–6.532]
Constipation				
Yes	36 (24)	14 (9.33)	1.14 [0.476–2.161]	0.600 [0.165–2.180]
No	71 (47.33)	29 (19.33)	1	1
Diarrhea				
Yes	60 (40)	24 (16)	1	1
No	47 (31.33)	19 (12.7)	0.957 [0.467–1.97]	1.253 [0.447–3.514]
Financial problem				
Yes	35 (23.33)	11 (7.33)	1	1
No	71 (47.33)	33 (22)	1.81 [0.787–4.191]	2.240 [0.711–7.064]
Need required				
Yes	59 (39.33)	30 (20)	0.67 [0.54–0.79]	0.85 [0.80–0.95]^*∗*^
No	26 (17.33)	35 (23.34	1	1
Level of satisfaction				
Low	72 (48)	25 (16.67)	0.73 [0.62–0.89]	0.82 [0.76–0.93]^*∗*^
High	34 (22.67)	19 (12.67)	1	1

^*∗*^Significant at 0.05 levels;  ^*∗∗*^significant at 0.01 levels.

## Data Availability

All data generated or analyzed during this study are included in this article.

## Abbreviations

AOR: Adjusted Odds Ratio
AKA: Also Known As
Ca: Cancer
CF: Cognitive functioning
COR: Crude Odds Ratio
EF: Emotional functioning
GHS: Global health status
HRQoL: Health related quality of life
QoL: Quality of life
OS: Overall Survival
ORR: Overall Radiographic Response
PF: Physical functioning
PFS: Progression-Free Survival
SF: Social functioning
UoGRH: University of Gondar Referral Hospital.
